# Genetic Diversity of Antimicrobial Resistance and Key Virulence Features in Two Extensively Drug-Resistant *Acinetobacter baumannii* Isolates

**DOI:** 10.3390/ijerph19052870

**Published:** 2022-03-01

**Authors:** Massimiliano Marazzato, Daniela Scribano, Meysam Sarshar, Francesca Brunetti, Silvia Fillo, Antonella Fortunato, Florigio Lista, Anna Teresa Palamara, Carlo Zagaglia, Cecilia Ambrosi

**Affiliations:** 1 Department of Public Health and Infectious Diseases, Sapienza University of Rome, 00185 Rome, Italy; massimiliano.marazzato@uniroma1.it (M.M.); daniela.scribano@uniroma1.it (D.S.); brunetti.1768585@studenti.uniroma1.it (F.B.); carlo.zagaglia@uniroma1.it (C.Z.); 2 Research Laboratories, Bambino Gesù Children’s Hospital, IRCCS, 00146 Rome, Italy; meysam.sarshar@uniroma1.it; 3 Scientific Department, Army Medical Center, 00184 Rome, Italy; silviafillo@gmail.com (S.F.); antonellafortunato75@gmail.com (A.F.); florigio.lista@esercito.difesa.it (F.L.); 4 Department of Infectious Diseases, Istituto Superiore di Sanità, 00161 Rome, Italy; annateresa.palamara@iss.it; 5 Department of Public Health and Infectious Diseases, Sapienza University of Rome, Laboratory Affiliated to Institute Pasteur Italia-Cenci Bolognetti Foundation, 00185 Rome, Italy; 6 Department of Human Sciences and Promotion of the Quality of Life, San Raffaele Open University, IRCCS, 00166 Rome, Italy

**Keywords:** *Acinetobacter baumannii*, microbial genomics, multidrug resistance, healthcare infections

## Abstract

In recent decades, *Acinetobacter baumannii* emerged as a major infective menace in healthcare settings due to scarce therapeutic options to treat infections. Therefore, undertaking genome comparison analyses of multi-resistant *A. baumannii* strains could aid the identification of key bacterial determinants to develop innovative anti-virulence approaches. Following genome sequencing, we performed a molecular characterization of key genes and genomic comparison of two *A. baumannii* strains, #36 and #150, with selected reference genomes. Despite a different antibiotic resistance gene content, the analyzed strains showed a very similar antibiogram profile. Interestingly, the lack of some important virulence determinants (i.e., *bap*, *ata* and *omp33*–*36*) did not abrogate their adhesive abilities to abiotic and biotic surfaces, as reported before; indeed, strains retained these capacities, although to a different extent, suggesting the presence of distinct vicarious genes. Conversely, secretion systems, lipopolysaccharide (LPS), capsule and iron acquisition systems were highly similar to *A. baumannii* reference strains. Overall, our analyses increased our knowledge on *A. baumannii* genomic content and organization as well as the genomic events occurring in nosocomial isolates to better fit into changing healthcare environments.

## 1. Introduction

In the last decade, the ubiquitous *Acinetobacter baumannii* has proven to be a great colonizer of healthcare surfaces and personnel. The increasing rate of antibiotic-resistant isolates creates difficulty to clinically manage *A. baumannii*-infected patients [1]. The most common infections caused by multidrug-resistant (MDR) *A. baumannii* strains include ventilator-associated pneumonia and bloodstream, urinary tract and skin and soft tissue infections, especially among critically ill patients in intensive care units (ICUs) [2,3,4]. Phenotypic and genotypic analyses demonstrated that there is a high degree of heterogeneity among healthcare-associated isolates [1,5,6]. Multilocus sequence typing (MLST) schemes were introduced to study the relationships among *A. baumannii* isolates [5,7]. This method of typing facilitates the discrimination of microbial isolates by comparing the sequences of housekeeping genes (*cpn60*, *fusA*, *gltA*, *pyrG*, *recA*, *rplB*, *rpoB*), thereby allowing the study of the distribution and spread of different sequence types (STs). It became clear that the occurrence and spread of different bacterial lineages followed a specific geographical distribution, leading to the classification of different *A. baumannii* international clones (ICs) across continents [2]. Therefore, globally distributed *A. baumannii* isolates underwent local clonal expansion due to their remarkable genomic plasticity, which is suited for acquiring and/or upregulating exogenous genes to quickly adapt to environmental/host changes [2,6,8,9]. Genome sequencing methods allowed us to highlight genetic elements and resistance genes characterizing specific *A. baumannii* isolates [10]. Therefore, the aim of this study is to analyze the genomes of two *A. baumannii* strains isolated in an Italian ICU, belonging to two different sequence types (STs), and compare them to reference strains used to study *A. baumannii* pathogenesis [11,12] in order to understand the adaptive changes occurring in each strain.

## 2. Results and Discussion

### 2.1. Genome Sequncing of A. baumannii Isolates and Phylogenetic Analysis of Selected Strains

Strains #36 and #150 belonging to ST78 and ST2 (https://pubmlst.org/organisms/acinetobacter-baumannii Accessed on 6 January 2022), respectively, were isolated from bronchial tracheal aspirate from patients admitted to the ICU of the University Hospital Policlinico Umberto I of Rome, Italy [13,14].

**Figure 1:**
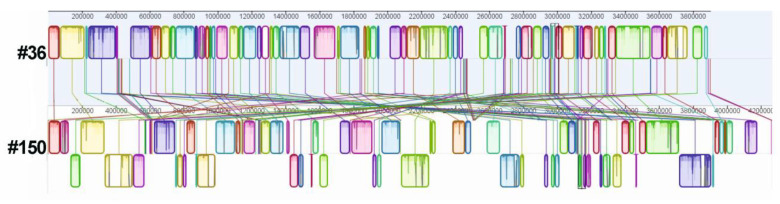
Genome alignment between strains #36 and #150, generated by the MAUVE aligner version 2.3.1. The progressive algorithm identifies stretches of matching nucleotides and selects locally collinear blocks (LCBs) that meet minimum weight criteria. The figure was generated by MAUVE viewer; homologous LCBs between genomes are represented by the same color and connected by lines. Inverted regions are depicted as blocks below the center line of the genome of strain #150.

The genome of each isolate was sequenced. Details of the sequencing and assembly are given in the Materials and Methods section. The assembly processes led to inferring a genome size of 3.90 and 4.26 Mbp with G+C contents of 39.0% and 38.9%, for strain #36 and strain #150, respectively. The whole-genome comparison between *A. baumannii* strains #36 and #150 is given in Figure 1.

**Figure 2:**
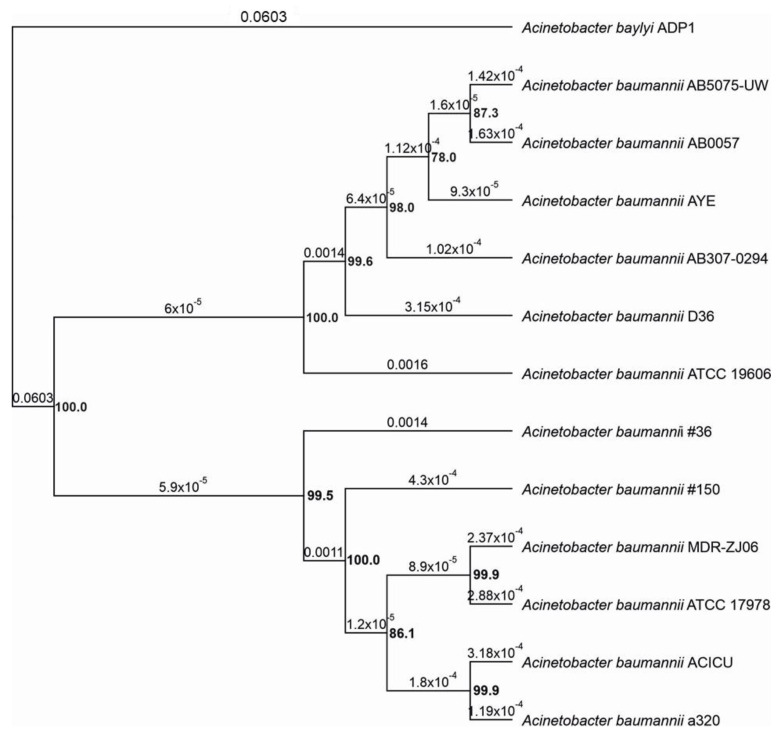
Core phylogeny tree of strains #36 and #150 in comparison with 10 available *A. baumannii* genomes. The numbers present on the branches of the tree represent the patristic distance used to estimate genetic divergence, while numbers in bold indicate bootstrap values relative to nodes. *Acinetobacter baylyi* ADP1 (NC_005966.1) was included in the analysis as an out-group.

The phylogenetic tree had been constructed on the base of the alignment of 1111 conserved genes, as determined by the BPGA pipeline [15], and neighbor-joining as an agglomerative method [16]. As shown in Figure 2, the phylogenetic reconstruction shows the presence of two distinct clusters, each of which incorporates reference sequences of different internal groups. Strains #36 and #150 fall within the second main cluster, showing more relatedness to the reference strain ATCC 17978.

### 2.2. Insertion Sequences (ISs) and Transposons

*A. baumannii* genomes harbor several mobile DNA elements often encompassing resistance genes [17]. To highlight the presence of ISs and transposons, the genomes of strains #36 and #150 were analyzed. Interestingly, strain #150 carries 3-fold more ISs than strain #36, among which IS*Aba1* is the prevalent one (Table 1). In *A. baumannii*, IS*Aba1* sequences are known major players for the transfer and expression of the carbapenem resistance gene [18]. Indeed, ISs are known to provide an outward-directed promoter (i.e., IS4, IS5, IS6) or hybrid promoter regions (i.e., IS30, IS21), thereby significantly affecting the expression of genes located downstream [19]. Additionally, we found a total of three unknown ISs in strain #150, encompassing the coding sequences of an unknown and two IS4 family transposases (Table 1).

Noteworthily, the two copies of ISVsa3 (IS91) carried within the genome of strain #36 showed a composite transposon containing the *sul*2 gene, encoding for resistance to sulfonamide, which was located 330 nt upstream of the gene. These elements are widely enclosed within plasmid elements, thereby favoring their spread by horizontal gene transfer and recombination events [20]. No phage sequences were found in either strain [21]. Therefore, the diversity of IS elements between these two *A. baumannii* isolates could account for the phenotypical differences previously reported in terms of antibiotic resistance, motility, biofilm-forming activity and host interaction mechanisms [13].

### 2.3. Resistance Genes

**Figure 3:**
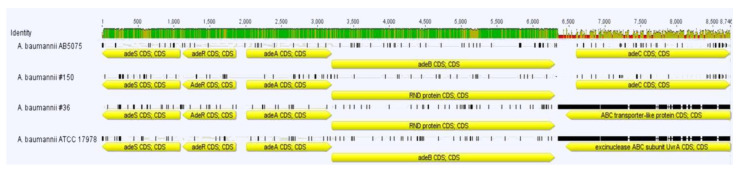
The gene structure of the *adeSRABC* locus in strains #36 and #150 in comparison with AB5075-UW (hereafter referred to as AB5075) and ATCC 17,978 reference strains. Predicted *ade* genes are displayed as arrows, whose direction is consistent with transcription direction. The analysis was performed with Geneious software version 7.1.3 (Biomatters, https://www.geneious.com accessed on 5 January 2022). The identity of compared sequences is shown. Green, full identity, yellow, <100 to 30% identity, red, <30% identity.

The antibiotic susceptibility profiles for both #36 and #150 isolates were made (Table 2). Interestingly, they display shared antibiotic resistance profiles, but strain #36 differs from strain #150 due to being susceptible to amikacin and tigecycline and intermediate susceptible (increased exposure) to cefepime (Table 2). Therefore, the presence of resistance genes harbored by #36 and #150 was analyzed by aligning the nucleotide sequences of these genes with the Comprehensive Antibiotic Resistance Database (CARD) [22]. Both strains displayed genes involved in aminoglycoside resistance, although there was a significant difference in the distribution of these resistance genes; strain #36 carries one acetyltransferase, *aac(6′)-Ian*, one nucleotidyltransferase, *ant(2’)-Ia*, and one phosphotransferase, *aph(3′)-1a*, whereas strain #150 possesses one acetyltransferase, *aac(3)-Ia*, and one adenyltransferase, *aadA2*. Noteworthily, in strain #36, the IS15DII and IS1006 were located 73 and 232 bp upstream of *ant(2′)-Ia* and *aac(6′)-Ian*, respectively, possibly contributing to resistance by gene overexpression. No 16S rRNA methylases (i.e., ArmA, RmtA, RmtB, RmtC and RmtD), known to confer an even higher level of resistance to all formulated aminoglycosides, were detected in either strain [23]. In addition, both #36 and #150 strains carry genes encoding tetrahydrofolate biosynthesic genes, *sul1* and *sul2*, respectively, conferring resistance to sulfonamide (Supplementary Table S1). As mentioned before, the *sul2* gene in strain #36 carries ISVsa3 [24], which possibly enhances its expression, thereby conferring high resistance to trimethoprim/sulfamethoxazole (Table 2). In this isolate, another ISVsa3 was found 217 nt upstream of the *floR* gene, conferring resistance to florfenicol, a veterinary analogue of chloramphenicol. In addition, both strains carry two variants of the extended-spectrum *ampC* cephalosporinase, named *Acinetobacter*-derived cephalosporinases (ADCs), ADC-6 in strain #36 and ADC-25 in strain #150 [25]. Finally, as a common strategy among multidrug-resistant *A. baumannii* isolates, both strains share a number of different efflux pump genes, displaying a high degree of identity (from 95.4 to 100%) with respect to those available from the NCBI GenBank database (Supplementary Table S1). These include the major facilitator superfamily (MFS) transporter genes *abaF* and *abaQ*, the multidrug and toxic efflux (MATE) *abeM* transporter gene, the small multidrug resistance (SMR) *abeS* transporter gene, the proteobacterial antimicrobial compound efflux (PACE) *aceI* transporter gene and the genes of the three resistance–nodulation–division (RND) superfamilies AdeABC, AdeFGH and AdeIJK, including the *adeRS* and *adeL* regulatory genes (Supplementary Table S1). Intriguingly, strain #36 lacks the *adeC* gene. Indeed, it carries an ABC transporter-like protein that does not match the nucleotide sequence of *adeC* (sequence identity of 36.7% with ATCC 17978) (Figure 3).

It has been reported that the presence of both *ant(2′)-Ia* and *aac(3′)-Ia* is correlated mostly with resistance to amikacin and kanamycin [26]. However, it is also known that amikacin resistance, compared to other aminoglycosides, is tightly associated to a functional *adeABC* [26]. Indeed, amikacin-resistant *A. baumannii* isolates showed a significant reduction in the MIC in the presence of 25 μg/mL efflux pump inhibitor [27]. The efflux pump AdeABC confers resistance to aminoglycosides, including amikacin, tetracyclines, fluoroquinolones, chloramphenicol and trimethoprim, and reduced susceptibility to tigecycline [28]. Additionally, in the presence of amikacin, it was reported that *adeB* was overexpressed [26,29]. Interestingly, previous studies revealed that *adeABC* overexpression was found to depend on functional amino acid mutations within conserved domains of AdeRS, the two-component system regulators [29]. The comparison of the nucleotide sequences of *adeS* and *adeR* between strain #150 and ATCC 17978 showed an identity of 97.56% and 98.52%, respectively. However, to search for key amino acid mutations that can alter the expression levels of both proteins, we inferred their amino acid sequences and compared them to the one from ATCC 17978, known for not overexpressing AdeABC [30]. AdeS from strain #150 displayed an identity of 97.48% (348/357) and a similarity of 99.44% (355/357) with AdeS from ATCC 17978, with seven conservative substitutions (data not shown). However, no known mutations that alter AdeABC expression level were found within the AdeS amino acid sequence of strain #150 [29]. Differently, AdeR displayed a high degree of identity and similarity (98.78% with 244/247 and 99.59% with 246/247, respectively) with AdeR from strain ATCC 17978. Of the only two mutations found, one is conservative (I→V), while the other at position 136 is not (A→V). Although we did not assess the levels of *adeABC* expression, we can speculate from the antibiogram profiles that strain #150 is fully amikacin and tigecycline resistant via the assistance of a functional AdeABC efflux pump (Table 2). Conversely, the lack of *adeC* in strain #36 forces the bacterium to compensate with other outer membrane proteins (OMPs) for the extrusion of both amikacin and tigecycline from the periplasmic space, thereby being less effective.

ADCs are chromosomally encoded class C β-lactamases, found in *A. baumannii* and other *Acinetobacter* spp., responsible for resistance to penicillins, cephalosporins and β-lactam/β-lactamase inhibitor combinations [17]. However, the activity of ADCs against the zwitterionic cephalosporin cefepime is debated, spanning from the absence of to some hydrolytic activity mediated by these β-lactamases [31,32,33]. Therefore, the intermediate susceptibility (increased exposure) of strain #36 carrying ADC-6 to cefepime is in agreement with the antibiogram profile of this strain, showing a low degree of cefepime resistance (Table 2). Vice versa, strain #150 is resistant to cefepime (MIC ≥ 64 mg/L), possessing the ADC-25 β-lactamase, which is the most prevalent among *A. baumannii* strains [34,35,36]. The *blaADC-25* nucleotide sequence alignment with available *A. baumannii* genomes revealed 100% identity with *blaADC-33*. The ADC-33 represents one of the most common β-lactamase variants whose amino acid mutations in the catalytic site allow for better cefepime binding and hydrolysis and hence it is responsible for cefepime resistance in *A. baumannii* [33]. It can be concluded that the high MIC value for cefepime in strain #150 is due to the presence of this efficient hydrolyzing enzyme.

Moreover, it has been reported that the *blaADC*-25 is often found downstream of the IS*Aba*1, which drives its overexpression [37,38]. Unfortunately, both ADC-6 and ADC-25 were located at one end of a contig and it was not possible to search for regulatory sequences upstream of the genes. Nevertheless, the activity of these enzymes together with the contribution of the efflux pumps as well as the reduced bacterial permeability could account for the antibiogram results.

### 2.4. Secretion Systems

A widely used strategy by Gram-negative bacteria to connect the inner compartment to the external environment is based on secretion systems (SSs). Among the eight types of SS, type 1 SS (T1SS), T2SS, T4SS and T6SS were identified in *A. baumannii* [2,17,39]. Due to the importance of SSs for *A. baumannii* for survival and virulence, the genes belonging to T1SS, T2SS, T4SS and T6SS were searched (Table 3). Among the genes encoding for T1SS, both #36 and #150 strains showed a high percentage of identity (ranging from 93.4 to 99.2%) with the reference strain AB5075. As the T1SS is involved in the export of important proteins involved in biofilm formation and maintenance, keeping a high level of homology of the permease/ATPase, type I secretion and hlyD genes should guarantee their proper functioning (Supplementary Table S1). An even higher degree of identity (ranging from 97.3 and 99.7%) with strain AB5075 was reached for T2SS for both strains, as reported for other Gram-negative bacteria [40], being fundamental for secreting folded proteins from the periplasm [2,41]. Conversely, the genes homologous to the Legionella/Coxiella T4SS (type IVB) were missing; only the icmH and rhs genes belonging to T4SS [42,43] were found in strain #36, with an identity of 98.4 and 80.1% with strains AB5075 and K09–14, respectively (Supplementary Table S1). Interestingly, in strain #150, icmH and the gene encoding type IV secretion system DNA-binding domain-containing protein were predicted to be located in the genomic sequences, whereas traC was plasmid encoded. Accordingly, the majority of the T4SS genes were located in plasmids in other *A. baumannii* strains while eight genes homologous to the Legionella/Coxiella T4SS have been found in a pathogenicity island (PAI) only in *A. baumannii* ATCC 17978 to date [44,45]. Therefore, the presence of two T4SS genes in strain #150 suggests that the genes move easily from a plasmid(s) to the bacterial chromosome. T5SS is based on the expression of monomeric or trimeric proteins; among the known subgroups, only the T5bSS (FhaB/C and CdiA/B) and T5cSS (Ata) subgroups were found in A. baumannii. Strain #36 carries fhaB, cdiB1 and cdiB2, encoding for FhaB, CdiB1 and CdiB2 (the components of the FhaB/FhaC and the CdiB-CdiA two-partner SS, respectively) [46,47]. These systems are involved in adhesion to host and bacterial cells and biofilm formation (Pérez et al., 2017, Roussin et al., 2019). However, the lack of cognate partner genes/proteins for both systems implies that they are not functional in strain #36. Interestingly, these genes were missing in strain #150, as previously reported [13] (Table 3; Supplementary Table S1). In addition, the ata gene, encoding a trimeric autotransporter involved in host cell adhesion and invasion, could not be found within the genomes of either strain. Nevertheless, both strains, and to a higher extent strain #150, exhibited a remarkable adhesion to the human A549 lung epithelial type II cell line (ATCC CCL185) [13]. Although it was shown that the ata gene is strongly conserved in 78% of *A. baumannii* isolates [48], its absence in both strains led us to conclude that they developed alternative systems to adhere effectively to biotic surfaces.

The T6SS is a very complex apparatus specifically evolved for injecting toxins into competitor bacteria in Acinetobacter spp. [2,52]. The 16 genes encoding the core structural T6SS proteins are clustered in a single genetic locus in Acinetobacter spp. [53]. Only 12 and 14 genes belonging to T6SS were found in strain #36 and #150, respectively, in that the genes encoding type VI secretion system tip proteins VgrG2 and VgrG3 could not be found in the genome of strain #36 (Supplementary Table S1). Interestingly, in addition to the tssJ gene, previously reported to be absent in Acinetobacter spp. [53], both strains lack the tssL and tolB genes, encoding a cytoplasmic protein bound to the inner membrane through a single transmembrane helix and the TonB-independent uptake of molecules, respectively [50]. Since it has been suggested that TssL interacts with TssM and contributes to T6SS functionality, its absence led us to speculate that T6SS may not work in either strain [50]. Accordingly, several genes encoding key T6SS proteins were shown to be missing in *A. baumannii* clinical strains [51,54,55,56]. Moreover, while the majority of the genes clustered together, the gene encoding type VI secretion system tip protein VgrG1 is located elsewhere in the chromosome of strain #150 (Supplementary Table S1). Despite several studies trying to elucidate the genetics, the proteins and the regulation of T6SS in A. baumannii, more work is needed to fully understand the role of this system for bacterial survival [46,56,57].

### 2.5. Outer Membrane Proteins, LPS and Capsule

Outer membrane proteins (Omps) are key bacterial components embedded within the outer membrane (OM) [58,59,60]. Omps carry out several roles in A. baumannii, including cellular permeability, antibiotic resistance, adherence to host cell and pathogenesis [2,4]. The genes encoding for major Omps (i.e., carO, csuD, dcaP, lptD, ompA, ompW, oprD) were identified in the genome of strain #36, with an identity that ranges from 81.8 to 99.3% with respect to the reference strain AB5075 (Supplementary Table S1). Interestingly, this strain lacks both oma87 and omp33–36 genes, the first involved in the biosynthesis and integrity of the outer membrane (OM), while Omp33-36 has a relevant role in fitness and virulence [2]. On the other side, strain #150 possesses the genes for major Omps with a variable percentage of identity (ranging from 78.9 to 100%) compared to strain AB5075, including oma87 and omp33–36, but lacks bamA and bap (Supplementary Table S1). Comparison of the biofilm formation and host cell adhesion ability between these strains showed that strain #36 was a greater biofilm producer, while strain #150 adhered to host cells to a higher extent [13]. The lack of Omp33–36 in strain #36 and Bap in strain #150 could reasonably account for these phenotypes.

*A. baumannii* produces a capsular polysaccharide encoded by a gene cluster referred to as the K locus, while the variable outer oligosaccharide of the LPS is encoded by the OC locus [60,61]. The K loci, KL3 and KL81, of strains #36 and #150, respectively, were highly conserved (ranging from 98.3 to 100%), in comparison to *A. baumannii* LUH3713 and ATCC 17978, respectively (Supplementary Table S1). Likewise, the OC loci, OCL1 and OCL3, encoding for the outer core oligosaccharides of the LPS in strain #36 and #150, respectively, were strongly similar to strain 85 and A1, with identity scores ranging from 98.3 to 100% (Supplementary Table S1).

### 2.6. Iron Scavengers

**Figure 4:**
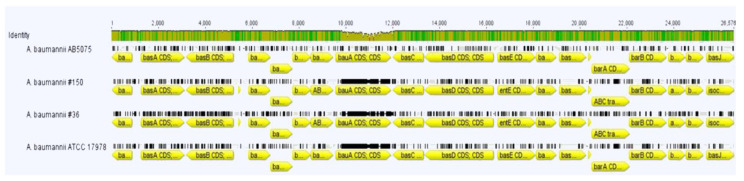
The gene structure of the acinetobactin locus in strains #36 and #150 in comparison with AB5075 and ATCC 17978 reference strains. Predicted *basA-J, barAB* and *bauA-E* genes are displayed as arrows, whose direction is consistent with transcription direction. The analysis was performed with Geneious software version 7.1.3 (Biomatters, https://www.geneious.com accessed on 5 January 2022, Auckland, New Zealand). The identity of compared sequences is shown. Green, full identity, yellow, <100 to 30% identity, red, <30% identity.

Being a cofactor for key enzymes, iron (Fe) is an essential nutrient for all living organisms. To fulfill its metabolic demand under aerobic environments, *A. baumannii* possesses redundant systems to capture iron in its oxidized ferric form (Fe(III)); among them, the most studied are those encoding the siderophores acinetobactin and pre-acinetobactin, and baumannoferrins A and B [2,62,63,64]. Due to the scarce bioavailability of this precious metal within hosts (i.e., natural “nutritional immunity”), these low molecular weight iron scavengers represent primary virulence factors for *A. baumannii* [3,65,66]. Therefore, gene clusters encoding for siderophores previously found in *A. baumannii* strains were searched in both #36 and #150 strains [66,67,68]. The whole cluster of genes responsible for the biosynthesis and transport of acinetobactin and pre-acinetobactin (i.e., basA-J, barAB and bauA-E) was present and highly conserved in both strains. The entA gene, encoding 2,3-dihydroxybenzoate-2,3-dehydrogenase, was frequently found outside the acinetobactin gene cluster in several *A. baumannii* strains and its genomic surroundings are extremely variable [69]. Regardless of its genomic location, entA encodes a key enzyme in the biosynthesis of acinetobactin [11,70]. In our strains, the genetic context of entA resembles the one found in ATCC 19606, in which entA and entB are located downstream of an uncharacterized molybdenum transport system, and upstream of the fur gene [70]. The percentage of nucleotide sequence identity with strain AB5075 ranged from 91.7 to 98.4% and 73.1 to 98.7% for strain #36 and #150, respectively, except for bauA, encoding the receptor for acinetobactin (Figure 4 and Supplementary Table S1). Indeed, this gene displayed a low nucleotide sequence identity with strain AB5075, with an identity percentage of 63.3 and 69.8% for strain #36 and #150, respectively. Interestingly, the amino acid sequences of BauA of *A. baumannii* ATCC 17978 and ATCC 19606 are 56.6% identical, suggesting that these typical OM TonB-dependent transporters evolved to became functionally different receptors, possibly to widen iron intake options [65,66]. Indeed, the comparison of the inferred amino acid sequences of BauA gave an identity of 99% (768/774) with ATCC 17978 and 58% (448/770) with ATCC 19606 for both strains. In addition, we found a short open reading frame of 156 nt, encoding a peptide of 51 amino acids, immediately upstream of the basG gene and transcribed in the same direction, in both strains (Supplementary Table S1). Unfortunately, no function could be inferred for this sequence, but it is widely found with a high degree of identity among different *A. baumannii* genomes. Therefore, this conservation led us to speculate that it might have some regulatory function.

The twelve genes encoding for the biosynthesis and transport of the secondary siderophore baumannoferrin (bfnA-L) were also searched. In both strains, the bfn gene cluster showed a remarkable percentage of identity (ranging from 97.3 to 99.1% for strain #36 and 97.7 to 99.8% for strain #150) to strain AB5075 (Supplementary Table S1), as well as other *A. baumannii* strains.

## 3. Materials and Methods

### 3.1. Bacterial Strains and Antimicrobial Susceptibility Testing (AST)

*A. baumannii* strains #36 and #150 were recovered from bronchial tracheal aspirates of ICU patients admitted to the University Hospital Policlinico Umberto I in Rome, Italy in December 2010 and November 2011, respectively [13,14]. AST was performed using a VITEK^®^2 system (bioMérieux, Italia S.p.A, Grassina, Italy) and interpreted according to European Committee on AST (EUCAST) criteria. The *A. baumannii* strains AB5075-UW and ATCC 17978 have been used as references for genomic comparisons (Table 4). The dataset generated in this study has been deposited with NCBI (Table 4).

### 3.2. Genome Sequencing

Genome sequencing was performed on an Illumina MiSeq platform using Nextera XT libraries kit v3 for sample preparation according to the manufacturer’s instructions (Illumina, San Diego, CA, USA). The sequence analysis allowed us to obtain a total of 2,936,352 high-quality paired-end reads for strain #36 and 3,079,124 for strain #150. The assembly of the genome relative to #36 and #150 resulted in 81 and 87 contigs, respectively. Each genomic assembly contained only contigs longer than 200 bp according to NCBI instructions (https://www.ncbi.nlm.nih.gov/genbank/wgsfaq/ Accessed on 25 February 2022). The quality of the original reads was evaluated using FASTQC [67] (available online at: http://www.bioinformatics.babraham.ac.uk/projects/fastqc/ Accessed on 15 January 2020). The reads were trimmed with Trimmomatic v. o.39 [69], and *de novo* assembled with SPAdes Assembler v.3.1.0. [72]. The numbers of estimated genes were 3685 and 4066, among which 69 and 82 were RNA genes for strain #36 and strain #150, respectively.

The core genome trees were constructed using the Bacterial Pan Genome Analysis (BPGA) software package with an 80% sequence identity cut-off [72]. To search the antibiotic resistance genes, the protein-coding sequences were aligned against the Comprehensive Antibiotic Resistance Database (CARD) [15]. The presence of specific genes related to surface proteins, efflux pumps, siderophores, resistance genes, LPS, capsule and secretion systems was determined by a BLAST search against reference sequences stored in various online databases at NCBI using Geneious software version 7.1.3 (Biomatters, https://www.geneious.com accessed on 5 January 2022), which generated multiple sequence images (Table 3 and Supplementary Table S1). In each case, 90% was considered as the threshold for both sequence coverage and sequence identity to determine positive results. ISs were identified using ISEscan [22]. Prediction and annotation of open reading frames were performed with Prokka v1.12 using the dedicated *Acinetobacter* database within the software [73]. The Progressive Mauve algorithm was used to create the whole genome alignment between the two *A. baumannii* strains #36 and #150, shown in Figure 1 [74]. All bioinformatics tools used in this study were run with default parameters.

### 3.3. Phylogenetic Analysis

The phylogenetic analysis of strains #36 and #150 in comparison with 10 available genomes was performed using IQ-TREE v.1.6.12 software [75]. The phylogenetic tree was based on a core phylogeny tree in comparison with 10 available *A. baumannii* genomes. The bootstrap modality (1000 bootstraps) was used to evaluate branch support. *Acinetobacter baylyi* ADP1 (NC_005966.1) was included in the analysis as an out-group.

## 4. Conclusions

The analyses performed in the present study allowed us to characterize the genomes of two *A. baumannii* strains isolated from in an Italian intensive care unit. The results presented herein corroborate other studies highlighting a constant genomic adaptation to better fit into the healthcare environment and/or the human host of *A. baumannii* isolates and be successful in terms of healthcare infections [2,20,30,76,77,78,79].

Despite a different antibiotic resistance gene content, the analyzed strains showed a very similar antibiogram profile (Table 2). Therefore, the antibiotic resistance paradigm, which relies on the acquisition of resistance genes, efflux pumps and IS elements whose insertion could dramatically change gene expression, also applies to strains #36 and #150. Additionally, these isolates previously showed a different degree of ability to adhere to abiotic and biotic surfaces [13]. Accordingly, the higher biofilm-forming ability of strain #36 in comparison with strain #150 could be explained by the absence of a conserved Bap protein in this strain; vice versa, the presence of Omp33–36 could account for the enhanced adhesion to pulmonary cells of strain #150 with respect to strain #36 [13]. Accordingly, previous studies revealed the absence of the *bap* gene in a number of *A. baumannii* isolates that displayed a reduced biofilm-forming ability [80,81].

The different SSs appeared well conserved with respect to the reference strain AB5075, possibly because mutations within these genes could dramatically affect *A. baumannii* pathogenicity and persistence [49]. Although several studies unraveled these systems, much remains largely understudied.

Genes encoding the LPS and the capsule showed a high percentage of identity when more reference strains were examined (Supplementary Table S1). Differently, the genes encoding acinetobactin and baumannoferrin were present and well conserved with respect to those of the reference strain AB5075. Due to the importance for in vivo fitness and virulence, iron acquisition systems are the best targets for designing conjugated antibiotics as well as iron-sequestering antimicrobials [82].

## Figures and Tables

**Table 1:** ISs found in the genomes of analyzed *A. baumannii* strains.

Insertion Sequences	Family	No. in #36	No. in #150
IS*Aba1*	IS4	2	21
IS*Aba12*	IS5	1	0
IS*Aba13*	IS5	0	1
IS*Aba14*	IS3	1	0
IS*Aba17*	IS66	0	2
IS*Aba19*	IS3	0	1
IS*Aba25*	IS66	2	0
IS*Aba27*	IS5	0	2
IS*Aba125*	IS30	0	3
ISAlw27	IS3	1	0
IS1006	IS6	2	0
IS26	IS6	0	1
ISVsa3	IS91	2	0
Unknown	IS4/IS481	0	3

**Table 2:** Antibiogram profile of both isolates, with minimal inhibitory concentrations (MICs) according to EUCAST.

Antibiotics	Strain	
	**#36**	**#150**
Amikacin	16 S	≥64 R
Amoxycillin/clavulanic acid	≥32 R	≥32 R
Ampicillin	≥32 R	≥32 R
Cefepime	16 IE	≥64 R
Cefotaxime	≥64 R	≥64 R
Ceftazidime	≥64 R	≥64 R
Ciprofloxacin	≥4 R	≥4 R
Colistin	≤0.5 S	≤0.5 S
Gentamicin	≥16 R	≥16 R
Imipenem	≥8 R	≥8 R
Piperacillin/Tazobactam	≥128 R	≥128 R
Tigecycline	≤0.5 S	≥8 R
Trimethoprim/Sulfamethoxazole	≥160 R	≥160 R

S, susceptible; R, resistant; IE, increased exposure (https://www.eucast.org/newsiandr/ Accessed on 20 December 2021).

**Table 3:** Main features of the different SSs found in *A. baumannii* strains #36 and #150.

Type of Secretion System (TSS)	Components	Translocation System	Genes Found in Both Strains #36 and #150	Reference
Type I secretion system (T1SS)	IM ABC transporter protein, a membrane fusion protein (MFP) and an OM protein	Single procedure (directly from cytoplasm to outside cell)	Type I SS permease/ATPase Type I secretion C-terminal target domain-containing protein HlyD family secretion protein 1 HlyD family secretion protein 2 HlyD family secretion protein 3 HlyD family secretion protein 4 HlyD family secretion protein 5 HlyD family type I secretion periplasmic adaptor subunit	[40]
Type II secretion system (T2SS)	IM SecYEG/Tat pathways, 15 general secretion pathway proteins (Gsp)	Double step procedure (Sec/Tat transfer the substrates of T2SS and T5SS across the inner membrane)	Type II secretion system F family protein *gspD* *gspE* *gspF* *gspG* *gspH* *gspI* *gspJ* *gspK* *gspL* *gspM* *gspN*	[49]
Type IV secretion system (T4SS)	Three type IVa ATPases, three IM proteins, a PP protein, two OM proteins, three surface/pilus proteins (tra and vir genes), eight genes homologous to the *Legionella/Coxiella* type IV virulence/secretion apparatus Dot/Icm	Single procedure (directly from cytoplasm into the outside of the cell)	*icmH* *traC* (#150) Type IV secretion system DNA-binding domain-containing protein (#150) Type IV secretion protein Rhs (#36)	[46,47,50,51]
Type V secretion system (T5SS)	An N-terminal Sec-dependent signal peptide, a central passenger domain and C-terminal β barrel	Two-partner and autotransporter	*abfhaB* (#36) *cdiB1* (#36) *cdiB2* (#36)	[2]
Type VI secretion system (T6SS)	Thirteen core components: membrane-spanning complex, baseplate components and priming protein TssA. VgrG-tipped Hcp tubule wrapped in the TssB/C sheath. No TssJ	Single procedure (directly from cytoplasm into the outside of the cell)	*tssA* *tssB* *tssC* *tssE* *tssF* *tssG* *tssH* *tssK* *tssL* *tssM* Type VI secretion system tip protein vgrg1 Type VI secretion system tip protein vgrg2 Type VI secretion system tip protein vgrg3 Type VI secretion system tube protein Hcp *tagF*	[49]

**Table 4:** Strains used for comparisons in this study.

Strain	Bioproject	Biosample	Accession No.	Reference
#36	PRJNA803948	SAMN25691074	-	This study
#150	PRJNA803948	SAMN25691075	-	This study
AB5075-UW	PRJNA224116	SAMN02894434	NZ_CP008706.1	[71]
ATCC 17978	PRJNA17477	SAMN02604331	NZ_CP053098.1	ATCC *

* American Type Culture Collection (Manassas, VA, USA).

## Data Availability

The Whole Genome Shotgun project of both strains has been deposited in GenBank (Table 4). In the meantime, the whole genome sequences are available upon request.

## Supplementary Materials

The following supporting information can be downloaded at: https://www.mdpi.com/article/10.3390/ijerph19052870/s1, Table S1: Main features, including contigs, genomic coordinates, coverage, identity percentage and reference strains from the analyses of the chromosomes of strains #36 and #150.

## References

[1] Bassetti M., Labate L., Russo C., Vena A., Giacobbe D.R. (2021). Therapeutic options for difficult-to-treat *Acinetobacter baumannii* infections: A 2020 perspective. Expert Opin. Pharmacother..

[2] Sarshar M., Behzadi P., Scribano D., Palamara A.T., Ambrosi C. (2021). *Acinetobacter baumannii*: An Ancient Commensal with Weapons of a Pathogen. Pathogens.

[3] Harding C.M., Hennon S.W., Feldman M.F. (2018). Uncovering the mechanisms of *Acinetobacter baumannii* virulence. Nat. Rev. Microbiol..

[4] Pompilio A., Scribano D., Sarshar M., Di Bonaventura G., Palamara A.T., Ambrosi C. (2021). Gram-Negative Bacteria Holding Together in a Biofilm: The *Acinetobacter baumannii* Way. Microorganisms.

[5] Diancourt L., Passet V., Nemec A., Dijkshoorn L., Brisse S. (2010). The population structure of *Acinetobacter baumannii*: Expanding multiresistant clones from an ancestral susceptible genetic pool. PLoS ONE.

[6] Ramirez M.S., Bonomo R.A., Tolmasky M.E. (2020). Carbapenemases: Transforming *Acinetobacter baumannii* into a Yet More Dangerous Menace. Biomolecules.

[7] Bartual S.G., Seifert H., Hippler C., Luzon M.A., Wisplinghoff H., Rodríguez-Valera F. (2005). Development of a multilocus sequence typing scheme for characterization of clinical isolates of *Acinetobacter baumannii*. J. Clin. Microbiol..

[8] Scribano D., Marzano V., Levi Mortera S., Sarshar M., Vernocchi P., Zagaglia C., Putignani L., Palamara A.T., Ambrosi C. (2019). Insights into the Periplasmic Proteins of *Acinetobacter baumannii* AB5075 and the Impact of Imipenem Exposure: A Proteomic Approach. Int. J. Mol. Sci..

[9] Ambrosi C., Scribano D., Sarshar M., Zagaglia C., Singer B.B., Palamara A.T. (2020). *Acinetobacter baumannii* Targets Human Carcinoembryonic Antigen-Related Cell Adhesion Molecules (CEACAMs) for Invasion of Pneumocytes. mSystems.

[10] Piran A., Fereshteh S., Badmasti F. (2021). Lessons from Comparative Genome Analysis of *Acinetobacter baumannii* Strains. Health Biotechnol. Biopharma.

[11] Antunes L.C., Imperi F., Carattoli A., Visca P. (2011). Deciphering the multifactorial nature of *Acinetobacter baumannii* pathogenicity. PLoS ONE.

[12] Jacobs A.C., Thompson M.G., Black C.C., Kessler J.L., Clark L.P., McQueary C.N., Gancz H.Y., Corey B.W., Moon J.K., Si Y. (2014). AB5075, a highly virulent isolate of *Acinetobacter baumannii*, as a model strain for the evaluation of pathogenesis and antimicrobial treatments. mBio.

[13] Ambrosi C., Scribano D., Aleandri M., Zagaglia C., Di Francesco L., Putignani L., Palamara A.T. (2017). *Acinetobacter baumannii* Virulence Traits: A Comparative Study of a Novel Sequence Type with Other Italian Endemic International Clones. Front. Microbiol..

[14] Ambrosi C., Aleandri M., Giordano A., Scribano D., Marazzato M., Zagaglia C., Conte M.P., Palamara A.T. (2016). Molecular characterisation of extensively drug-resistant *Acinetobacter baumannii*: First report of a new sequence type in Italy. J. Glob. Antimicrob. Resist..

[15] Chaudhari N.M., Gupta V.K., Dutta C. (2016). BPGA-an ultra-fast pan-genome analysis pipeline. Sci. Rep..

[16] Bryant D., Moulton V. (2004). Neighbor-net: An agglomerative method for the construction of phylogenetic networks. Mol. Biol. Evol..

[17] Ayoub Moubareck C., Hammoudi Halat D. (2020). Insights into *Acinetobacter baumannii*: A Review of Microbiological, Virulence, and Resistance Traits in a Threatening Nosocomial Pathogen. Antibiotics.

[18] Turton J.F., Ward M.E., Woodford N., Kaufmann M.E., Pike R., Livermore D.M., Pitt T.L. (2006). The role of ISAba1 in expression of OXA carbapenemase genes in *Acinetobacter baumannii*. FEMS Microbiol. Lett..

[19] Vandecraen J., Chandler M., Aertsen A., Van Houdt R. (2017). The impact of insertion sequences on bacterial genome plasticity and adaptability. Crit. Rev. Microbiol..

[20] Snitkin E.S., Zelazny A.M., Montero C.I., Stock F., Mijares L., Murray P.R., Segre J.A. (2011). Genome-wide recombination drives diversification of epidemic strains of *Acinetobacter baumannii*. Proc. Natl. Acad. Sci. USA.

[21] Fouts D.E. (2006). Phage_Finder: Automated identification and classification of prophage regions in complete bacterial genome sequences. Nucleic Acids Res..

[22] Alcock B.P., Raphenya A.R., Lau T.T.Y., Tsang K.K., Bouchard M., Edalatmand A., Huynh W., Nguyen A.V., Cheng A.A., Liu S. (2020). CARD 2020: Antibiotic resistome surveillance with the comprehensive antibiotic resistance database. Nucleic Acids Res..

[23] Liu Z., Ling B., Zhou L. (2015). Prevalence of 16S rRNA methylase, modifying enzyme, and extended-spectrum beta-lactamase genes among *Acinetobacter baumannii* isolates. J. Chemother..

[24] He T., Wang R., Liu D., Walsh T.R., Zhang R., Lv Y., Ke Y., Ji Q., Wei R., Liu Z. (2019). Emergence of plasmid-mediated high-level tigecycline resistance genes in animals and humans. Nat. Microbiol..

[25] Hujer K.M., Hamza N.S., Hujer A.M., Perez F., Helfand M.S., Bethel C.R., Thomson J.M., Anderson V.E., Barlow M., Rice L.B. (2005). Identification of a new allelic variant of the *Acinetobacter baumannii* cephalosporinase, ADC-7 beta-lactamase: Defining a unique family of class C enzymes. Antimicrob. Agents Chemother..

[26] Sheikhalizadeh V., Hasani A., Ahangarzadeh Rezaee M., Rahmati-Yamchi M., Hasani A., Ghotaslou R., Goli H.R. (2017). Comprehensive study to investigate the role of various aminoglycoside resistance mechanisms in clinical isolates of *Acinetobacter baumannii*. J. Infect. Chemother. Off. J. Jpn. Soc. Chemother..

[27] Ostadi Y., Rezai A.A., Moghadampour M., Faghri J. (2019). The involvement of drug efflux system in amikacin resistance of multiple drug resistant Acintobacter baumannii isolates in Isfahan, Iran. J. Med. Bacteriol..

[28] Abdi S.N., Ghotaslou R., Ganbarov K., Mobed A., Tanomand A., Yousefi M., Asgharzadeh M., Kafil H.S. (2020). *Acinetobacter baumannii* Efflux Pumps and Antibiotic Resistance. Infect. Drug Resist..

[29] Yoon E.-J., Courvalin P., Grillot-Courvalin C. (2013). RND-type efflux pumps in multidrug-resistant clinical isolates of *Acinetobacter baumannii*: Major role for AdeABC overexpression and AdeRS mutations. Antimicrob. Agents Chemother..

[30] Adams M.D., Goglin K., Molyneaux N., Hujer K.M., Lavender H., Jamison J.J., MacDonald I.J., Martin K.M., Russo T., Campagnari A.A. (2008). Comparative genome sequence analysis of multidrug-resistant *Acinetobacter baumannii*. J. Bacteriol..

[31] Pérez A., Pérez-Llarena F.J., García P., Kerff F., Beceiro A., Galleni M., Bou G. (2014). New mutations in ADC-type β-lactamases from Acinetobacter spp. affect cefoxitin and ceftazidime hydrolysis. J. Antimicrob. Chemother..

[32] Tian G.-B., Adams-Haduch J.M., Taracila M., Bonomo R.A., Wang H.-N., Doi Y. (2011). Extended-spectrum AmpC cephalosporinase in *Acinetobacter baumannii*: ADC-56 confers resistance to cefepime. Antimicrob. Agents Chemother..

[33] Rodríguez-Martínez J.-M., Nordmann P., Ronco E., Poirel L. (2010). Extended-spectrum cephalosporinase in *Acinetobacter baumannii*. Antimicrob. Agents Chemother..

[34] Wareth G., Linde J., Nguyen N.H., Nguyen T.N., Sprague L.D., Pletz M.W., Neubauer H. (2021). WGS-Based Analysis of Carbapenem-Resistant *Acinetobacter baumannii* in Vietnam and Molecular Characterization of Antimicrobial Determinants and MLST in Southeast Asia. Antibiotics.

[35] Chapartegui-González I., Lázaro-Díez M., Redondo-Salvo S., Navas J., Ramos-Vivas J. (2021). Antimicrobial Resistance Determinants in Genomes and Plasmids from *Acinetobacter baumannii* Clinical Isolates. Antibiotics.

[36] Hujer A.M., Hujer K.M., Leonard D.A., Powers R.A., Wallar B.J., Mack A.R., Taracila M.A., Rather P.N., Higgins P.G., Prati F. (2021). A comprehensive and contemporary “snapshot” of β-lactamases in carbapenem resistant *Acinetobacter baumannii*. Diagn. Microbiol. Infect. Dis..

[37] Dortet L., Bonnin R.A., Bernabeu S., Escaut L., Vittecoq D., Girlich D., Imanci D., Fortineau N., Naas T. (2016). First occurrence of OXA-72-producing *Acinetobacter baumannii* in Serbia. Antimicrob. Agents Chemother..

[38] Rossi I., Royer S., Ferreira M., Braga I.A., Campos P., Batistão D., Fuga B., Cerdeira L., Lincopan N., Gontijo-Filho P.P. (2021). Novel ST1465/CC216 Nosocomial Lineage of Carbapenem-Resistant *Acinetobacter baumannii* Harboring an Unusual Plasmid Carrying bla NDM-1 Gene. Microb. Drug Resist..

[39] Elhosseiny N.M., Attia A.S. (2018). Acinetobacter: An emerging pathogen with a versatile secretome. Emerg. Microbes Infect..

[40] Pena R.T., Blasco L., Ambroa A., González-Pedrajo B., Fernández-García L., López M., Bleriot I., Bou G., García-Contreras R., Wood T.K. (2019). Relationship between quorum sensing and secretion systems. Front. Microbiol..

[41] Korotkov K.V., Sandkvist M., Hol W.G. (2012). The type II secretion system: Biogenesis, molecular architecture and mechanism. Nat. Rev. Microbiol..

[42] Koskiniemi S., Lamoureux J.G., Nikolakakis K.C., de Roodenbeke C.t.K., Kaplan M.D., Low D.A., Hayes C.S. (2013). Rhs proteins from diverse bacteria mediate intercellular competition. Proc. Natl. Acad. Sci. USA.

[43] Zusman T., Feldman M., Halperin E., Segal G. (2004). Characterization of the icmH and icmF genes required for Legionella pneumophila intracellular growth, genes that are present in many bacteria associated with eukaryotic cells. Infect. Immun..

[44] Smith M.G., Gianoulis T.A., Pukatzki S., Mekalanos J.J., Ornston L.N., Gerstein M., Snyder M. (2007). New insights into *Acinetobacter baumannii* pathogenesis revealed by high-density pyrosequencing and transposon mutagenesis. Genes Dev..

[45] Liu C.-C., Kuo H.-Y., Tang C.Y., Chang K.-C., Liou M.-L. (2014). Prevalence and mapping of a plasmid encoding a type IV secretion system in *Acinetobacter baumannii*. Genomics.

[46] Pérez A., Merino M., Rumbo-Feal S., Álvarez-Fraga L., Vallejo J.A., Beceiro A., Ohneck E.J., Mateos J., Fernández-Puente P., Actis L.A. (2017). The FhaB/FhaC two-partner secretion system is involved in adhesion of *Acinetobacter baumannii* AbH12O-A2 strain. Virulence.

[47] Roussin M., Rabarioelina S., Cluzeau L., Cayron J., Lesterlin C., Salcedo S.P., Bigot S. (2019). Identification of a Contact-Dependent Growth Inhibition (CDI) System That Reduces Biofilm Formation and Host Cell Adhesion of *Acinetobacter baumannii* DSM30011 Strain. Front. Microbiol..

[48] Weidensdorfer M., Ishikawa M., Hori K., Linke D., Djahanschiri B., Iruegas R., Ebersberger I., Riedel-Christ S., Enders G., Leukert L. (2019). The Acinetobacter trimeric autotransporter adhesin Ata controls key virulence traits of *Acinetobacter baumannii*. Virulence.

[49] Weber B.S., Kinsella R.L., Harding C.M., Feldman M.F. (2017). The secrets of Acinetobacter secretion. Trends Microbiol..

[50] Ruiz F.M., Lopez J., Ferrara C.G., Santillana E., Espinosa Y.R., Feldman M.F., Romero A. (2020). Structural characterization of TssL from *Acinetobacter baumannii*: A key component of the type VI secretion system. J. Bacteriol..

[51] Wright M.S., Haft D.H., Harkins D.M., Perez F., Hujer K.M., Bajaksouzian S., Benard M.F., Jacobs M.R., Bonomo R.A., Adams M.D. (2014). New insights into dissemination and variation of the health care-associated pathogen *Acinetobacter baumannii* from genomic analysis. mBio.

[52] Russell A.B., Hood R.D., Bui N.K., LeRoux M., Vollmer W., Mougous J.D. (2011). Type VI secretion delivers bacteriolytic effectors to target cells. Nature.

[53] Weber B.S., Miyata S.T., Iwashkiw J.A., Mortensen B.L., Skaar E.P., Pukatzki S., Feldman M.F. (2013). Genomic and functional analysis of the type VI secretion system in Acinetobacter. PLoS ONE.

[54] Kim J., Lee J.-Y., Lee H., Choi J.Y., Kim D.H., Wi Y.M., Peck K.R., Ko K.S. (2017). Microbiological features and clinical impact of the type VI secretion system (T6SS) in *Acinetobacter baumannii* isolates causing bacteremia. Virulence.

[55] Meumann E.M., Anstey N.M., Currie B.J., Piera K.A., Kenyon J.J., Hall R.M., Davis J.S., Sarovich D.S. (2019). Genomic epidemiology of severe community-onset *Acinetobacter baumannii* infection. Microb. Genom..

[56] Traglia G., Chiem K., Quinn B., Fernandez J.S., Montaña S., Almuzara M., Mussi M.A., Tolmasky M.E., Iriarte A., Centrón D. (2018). Genome sequence analysis of an extensively drug-resistant *Acinetobacter baumannii* indigo-pigmented strain depicts evidence of increase genome plasticity. Sci. Rep..

[57] Park J.S., Lee W.C., Yeo K.J., Ryu K.S., Kumarasiri M., Hesek D., Lee M., Mobashery S., Song J.H., Kim S.I. (2012). Mechanism of anchoring of OmpA protein to the cell wall peptidoglycan of the gram-negative bacterial outer membrane. FASEB J. Off. Publ. Fed. Am. Soc. Exp. Biol..

[58] Nie D., Hu Y., Chen Z., Li M., Hou Z., Luo X., Mao X., Xue X. (2020). Outer membrane protein A (OmpA) as a potential therapeutic target for *Acinetobacter baumannii* infection. J. Biomed. Sci..

[59] Ambrosi C., Pompili M., Scribano D., Zagaglia C., Ripa S., Nicoletti M. (2012). Outer membrane protein A (OmpA): A new player in shigella flexneri protrusion formation and inter-cellular spreading. PLoS ONE.

[60] Singh J.K., Adams F.G., Brown M.H. (2019). Diversity and function of capsular polysaccharide in *Acinetobacter baumannii*. Front. Microbiol..

[61] Kenyon J.J., Nigro S.J., Hall R.M. (2014). Variation in the OC locus of *Acinetobacter baumannii* genomes predicts extensive structural diversity in the lipooligosaccharide. PLoS ONE.

[62] Runci F., Gentile V., Frangipani E., Rampioni G., Leoni L., Lucidi M., Visaggio D., Harris G., Chen W., Stahl J. (2019). Contribution of active iron uptake to *Acinetobacter baumannii* pathogenicity. Infect. Immun..

[63] Sheldon J.R., Skaar E.P. (2020). *Acinetobacter baumannii* can use multiple siderophores for iron acquisition, but only acinetobactin is required for virulence. PLoS Pathog..

[64] Shapiro J.A., Wencewicz T.A. (2016). Acinetobactin isomerization enables adaptive iron acquisition in *Acinetobacter baumannii* through pH-triggered siderophore swapping. ACS Infect. Dis..

[65] Moynié L., Serra I., Scorciapino M.A., Oueis E., Page M.G., Ceccarelli M., Naismith J.H. (2018). Preacinetobactin not acinetobactin is essential for iron uptake by the BauA transporter of the pathogen *Acinetobacter baumannii*. eLife.

[66] Klebba P.E., Newton S.M., Six D.A., Kumar A., Yang T., Nairn B.L., Munger C., Chakravorty S. (2021). Iron Acquisition Systems of Gram-negative Bacterial Pathogens Define TonB-Dependent Pathways to Novel Antibiotics. Chem. Rev..

[67] Andrews S., FASTQC (2010). A quality control tool for high throughput sequence data. https://www.bioinformatics.babraham.ac.uk/projects/fastqc/.

[68] Bolger A.M., Lohse M., Usadel B. (2014). Trimmomatic: A flexible trimmer for Illumina sequence data. Bioinformatics.

[69] Hasan T., Choi C.H., Oh M.H. (2015). Genes Involved in the Biosynthesis and Transport of Acinetobactin in *Acinetobacter baumannii*. Genom. Inform..

[70] Penwell W.F., Arivett B.A., Actis L.A. (2012). The *Acinetobacter baumannii* entA gene located outside the acinetobactin cluster is critical for siderophore production, iron acquisition and virulence. PLoS ONE.

[71] Gallagher L.A., Ramage E., Weiss E.J., Radey M., Hayden H.S., Held K.G., Huse H.K., Zurawski D.V., Brittnacher M.J., Manoil C. (2015). Resources for Genetic and Genomic Analysis of Emerging Pathogen *Acinetobacter baumannii*. J. Bacteriol..

[72] Bankevich A., Nurk S., Antipov D., Gurevich A.A., Dvorkin M., Kulikov A.S., Lesin V.M., Nikolenko S.I., Pham S., Prjibelski A.D. (2012). SPAdes: A new genome assembly algorithm and its applications to single-cell sequencing. J. Comput. Biol..

[73] Xie Z., Tang H. (2017). ISEScan: Automated identification of insertion sequence elements in prokaryotic genomes. Bioinformatics.

[74] Seemann T. (2014). Prokka: Rapid prokaryotic genome annotation. Bioinformatics.

[75] Darling A.C., Mau B., Blattner F.R., Perna N.T. (2004). Mauve: Multiple alignment of conserved genomic sequence with rearrangements. Genome Res..

[76] Minh B.Q., Schmidt H.A., Chernomor O., Schrempf D., Woodhams M.D., Von Haeseler A., Lanfear R. (2020). IQ-TREE 2: New models and efficient methods for phylogenetic inference in the genomic era. Mol. Biol. Evol..

[77] Antunes L.C., Visca P., Towner K.J. (2014). *Acinetobacter baumannii*: Evolution of a global pathogen. Pathog. Dis..

[78] Imperi F., Antunes L.C., Blom J., Villa L., Iacono M., Visca P., Carattoli A. (2011). The genomics of *Acinetobacter baumannii*: Insights into genome plasticity, antimicrobial resistance and pathogenicity. IUBMB Life.

[79] Yakkala H., Samantarrai D., Gribskov M., Siddavattam D. (2019). Comparative genome analysis reveals niche-specific genome expansion in *Acinetobacter baumannii* strains. PLoS ONE.

[80] Badmasti F., Siadat S.D., Bouzari S., Ajdary S., Shahcheraghi F. (2015). Molecular detection of genes related to biofilm formation in multidrug-resistant *Acinetobacter baumannii* isolated from clinical settings. J. Med. Microbiol..

[81] Jalal D., Elzayat M.G., Diab A.A., El-Shqanqery H.E., Samir O., Bakry U., Hassan R., Elanany M., Shalaby L., Sayed A.A. (2021). Deciphering Multidrug-Resistant *Acinetobacter baumannii* from a Pediatric Cancer Hospital in Egypt. Msphere.

[82] Ribeiro M., Simões M. (2019). Advances in the antimicrobial and therapeutic potential of siderophores. Environ. Chem. Lett..

